# Molecular identification of *Comstockaspis perniciosa* and its associated *Aphytis* parasitoid in apple orchards of Kazakhstan

**DOI:** 10.3389/finsc.2026.1960516

**Published:** 2026-10-07

**Authors:** Alibek Makhambetov, Valeriya Kostyukova, Gulnur Zholdasbek, Zarina Dairbekova, Bakyt Dulat, Alexandr Pozharskiy, Gulshariya Kairova, Dilyara Gritsenko

**Affiliations:** 1 Laboratory of Molecular Biology, Institute of Plant Biology and Biotechnology, Almaty, Kazakhstan; 2 Department of Molecular Biology and Genetics, Al Farabi Kazakh National University, Almaty, Kazakhstan; 3 Kazakh National Agrarian Research University, Almaty, Kazakhstan; 4 Research Center AgriBioTech, Almaty, Kazakhstan

**Keywords:** 28S rRNA, *Aphytis*, apple orchards, *comstockaspis perniciosa*, Kazakhstan, next generation sequencing, parasitoid, quarantine pests

## Abstract

**Background:**

*Comstockaspis perniciosa* (Comstock, 1881) is one of the most destructive quarantine pests of fruit crops, posing a serious threat to commercial orchards and natural populations of wild apple trees. Despite its widespread distribution, information on the molecular genetic characteristics of this species in Central Asia remains limited.

**Methods:**

The aim of this study was to characterize *C. perniciosa* collected from commercial orchards in southeastern Kazakhstan based on external morphological observations and molecular data, determine the phylogenetic position of the examined specimens using 28S rRNA gene sequences, and detect associated parasitoids using high-throughput sequencing technologies. During the growing season in 2025, surveys were conducted in ten commercial orchards, including sites located near natural populations of wild apple trees.

**Results:**

Species-level identification of the collected specimens was confirmed by analysis of the 28S rRNA gene. All sequences clustered with reference *C. perniciosa* sequences, confirming species identity. Analysis of the high-throughput sequencing data additionally revealed sequences of parasitoids belonging to the genus *Aphytis* (Hymenoptera: Aphelinidae) in ten samples, indicating the presence of natural enemies associated with *C. perniciosa*.

**Conclusions:**

These findings expand current knowledge of the molecular characteristics of *C. perniciosa* in Central Asia and demonstrate the potential of NGS (Next Generation Sequencing) technologies for the simultaneous detection of quarantine pests and their associated entomophagous natural enemies within phytosanitary monitoring programs.

## 1 Introduction

*Comstockaspis perniciosa* (Comstock, 1881) (Hemiptera: Diaspididaeis one of the most destructive invasive phytophagous pests of temperate fruit crops (1). Its broad host range, high ecological plasticity, and considerable invasive potential contribute to the substantial economic losses caused by *C. perniciosa* (2, 3). Local adaptation to particular host plant genotypes has been suggested for some armored scale insects (4).

*C. perniciosa* is currently distributed on nearly all continents and infests more than 125 species of woody plants and shrubs, causing the greatest economic damage to apple, pear, peach, plum, and other fruit crops (5, 6). The insect damages bark, shoots, and fruits, resulting in reduced tree vigor, dieback of branches, decreased yield, and deterioration of fruit quality. An intensive infestations may cause young trees to die prematurely (1, 3). On apple fruits, a characteristic symptom of infestation is the formation of reddish halos around feeding sites, which significantly reduces their market value (7).

The spread of *C. perniciosa* is of particular concern in Kazakhstan because the country is the center of origin of *Malus sieversii* (Ledeb.) M.Roem., the primary wild progenitor of the cultivated apple, as well as the rare species *Malus niedzwetzkyana* Dieck (8). Populations of these wild apple species represent a unique global genetic resource of exceptional importance for biodiversity conservation and fruit crop breeding (9, 10). The close proximity of commercial, private, and abandoned orchards to natural apple forests creates a persistent risk of quarantine phytophagous pests spreading between agricultural and natural ecosystems. Abandoned orchards may serve as long-term reservoirs for *C. perniciosa* populations, maintaining infestation foci and facilitating further dispersal, including to wild *Malus* species (3, 11, 12). Therefore, monitoring and molecular identification of *C. perniciosa* populations are important not only for the phytosanitary management of fruit orchards but also for assessing potential risks to natural ecosystems.

In addition to studying the pest itself, investigation of its natural enemies is of considerable importance. Among the most effective parasitoids of *C. perniciosa* are species of the genus *Aphytis* Howard (Hymenoptera: Aphelinidae), which are widely used in biological control programs targeting armored scale insects (13). Recent advances in sequencing technologies have greatly expanded opportunities to investigate such trophic interactions. In the present study, analysis of long-read sequences generated using Oxford Nanopore Technologies revealed not only sequences of *Comstockaspis perniciosa* but also sequences belonging to representatives of the genus *Aphytis*. This finding indicates the presence of parasitoid DNA in the analyzed material and demonstrates the potential of long-read sequencing for the simultaneous detection of phytophagous pests and their associated parasites or symbionts.

Despite the widespread occurrence of *C. perniciosa*, information on the molecular genetic characteristics of its populations in Central Asia remains limited, and data on its associated parasitoid complexes are scarce. The aim of the present study was to identify *Comstockaspis perniciosa* specimens collected from apple trees using 28S rRNA gene sequence analysis, record their external morphological characteristics during specimen examination, determine their phylogenetic position within the family Diaspididae, and identify sequences of parasitoids of the genus *Aphytis* detected during the analysis of Oxford Nanopore Technologies sequencing reads.

## 2 Materials and methods

### 2.1 Sample collection and preliminary morphological identification

Samples were collected during the 2025 growing season from commercial apple orchards in southeastern Kazakhstan. A total of ten sampling sites were surveyed (**Table 1**), including orchards located in close proximity to natural populations of wild apple trees. The sampling sites were selected based on the geographic distribution and availability of commercial apple orchards within the study area. Insects were collected using a random sampling approach. During the survey, 56 armored scale insect samples were collected.

**Table 1:** Sampling locations.

No. on map	Sampling location	Coordinates
1	Baiseit Village (Apple and Peach orchards)	43.58694528 78.33305795
2		43.58222306 78.34028017
3		43.5866675 78.33889128
4		43.58333417 78.33889128
5		43.57083417 78.33889128
6		43.58888972 78.33861351
7		43.5841675 78.33805795
8	Bakhar Village, Chunja District	43.60444526 79.41305794
9	Zharkent, Panfilov District	44.17722301 80.13666907
10	Enbekshikazakh District	43.58250083 78.35389128

Preliminary identification of *Comstockaspis perniciosa* was performed based on external morphological characteristics using published diagnostic descriptions and identification keys (14, 15). Adult females and first-instar crawlers were examined using a Leica Ivesta 3 stereomicroscope (Leica Microsystems, Germany). Species-level identification was subsequently confirmed by 28S rRNA gene sequence analysis.

### 2.2 Taxonomic identification using nanopore sequencing

Genomic DNA was extracted using the Monarch^®^ Spin gDNA Extraction Kit (New England Biolabs, USA) according to the manufacturer’s instructions. DNA concentration and purity were assessed using a NanoDrop One spectrophotometer (Thermo Fisher Scientific, USA).

For the molecular characterization of *Comstockaspis perniciosa*, the nuclear 28S ribosomal RNA gene (28S rDNA) was selected as the genetic marker. PCR amplification was performed using the primer pair 28S3a (AGTACGTGAAACCGTTCAGG) and 28Sb (TCGGAAGGAACCAGCTAC) (16). PCR reactions were carried out in a total volume of 25 µL containing 1 U Taq DNA Polymerase (New England Biolabs, USA), 1× Taq buffer, 0.2 mM dNTPs, 0.2 µM of each primer, and 20 ng of template DNA. Amplification was performed under the following conditions: initial denaturation at 95 °C for 1 min, followed by amplification cycles consisting of denaturation at 95 °C, annealing at 48 °C for 1 min, and extension at 72 °C for 2 min, with a final extension at 72 °C for 7 min.

Sequencing libraries were prepared using the Rapid Barcoding Kit SQK-RBK114.96 (Oxford Nanopore Technologies, Oxford, UK) according to the manufacturer’s recommendations. High-throughput sequencing was performed on a PromethION P2 Solo platform (Oxford Nanopore Technologies, UK). Raw signal data were basecalled in MinKNOW (v25.05.14) using the integrated Dorado basecaller (v7.9.8), and reads with mean Phred quality scores below 12 were removed using NanoFilt v2.8.0 (17). Quality-filtered reads were subsequently analyzed with NGSpeciesID v0.3.1 (18), which clusters similar nanopore reads and generates *de novo* consensus sequences for the identified clusters. The resulting consensus sequences were further polished using Medaka v2.0.1 to reduce the residual error rate characteristic of Oxford Nanopore sequencing.

All polished consensus sequences generated for each barcode were compared against the NCBI nucleotide database using the BLASTn algorithm. Taxonomic assignments were based on the most informative matches, taking into account query coverage, percentage sequence identity, and alignment significance.

Separate reference databases of 28S rRNA gene sequences were compiled for the families Diaspididae and Aphelinidae using publicly available GenBank records. Quality-filtered reads from barcodes containing a consensus sequence assigned to Aphelinidae were first mapped against the Diaspididae 28S rRNA reference database using minimap2 (19) in Oxford Nanopore read mode.

Reads that did not map to the Diaspididae reference database were subsequently mapped against the Aphelinidae 28S rRNA reference database. Reads successfully aligned to the latter were classified as Aphelinidae-associated, whereas reads that failed to align to either family-specific reference database were classified as unassigned. Alignment files were processed using SAMtools v1.23.1 (20), after which the numbers of reads assigned to Diaspididae, Aphelinidae, and the unassigned fraction were calculated for each barcode. The relative abundance of each read category was expressed as a percentage of the total number of reads. Data visualization was performed in Python v3.14.3 using the pandas v3.0.2 (20), and Matplotlib v3.10.8 (21) libraries, generating 100% normalized horizontal stacked bar charts.

For phylogenetic analysis, reference sequences were retrieved from the public NCBI database. Multiple sequence alignment was performed using the MAFFT algorithm (22) implemented in UGENE v50.0 0 (23). Phylogenetic relationships were inferred in MEGA 11 (24) using the Maximum Composite Likelihood model with 1,000 bootstrap replicates, and the resulting phylogenetic tree was further edited and visualized in FigTree v1.4.4.

## 3 Results

During the field survey of commercial apple orchards in southeastern Kazakhstan, ten sampling sites were examined (**Figure 1**), several of which were located in close proximity to natural populations of wild fruit trees. A total of 56 armored scale insect samples were collected and subsequently used for morphological and molecular analyses.

**Figure 1:**
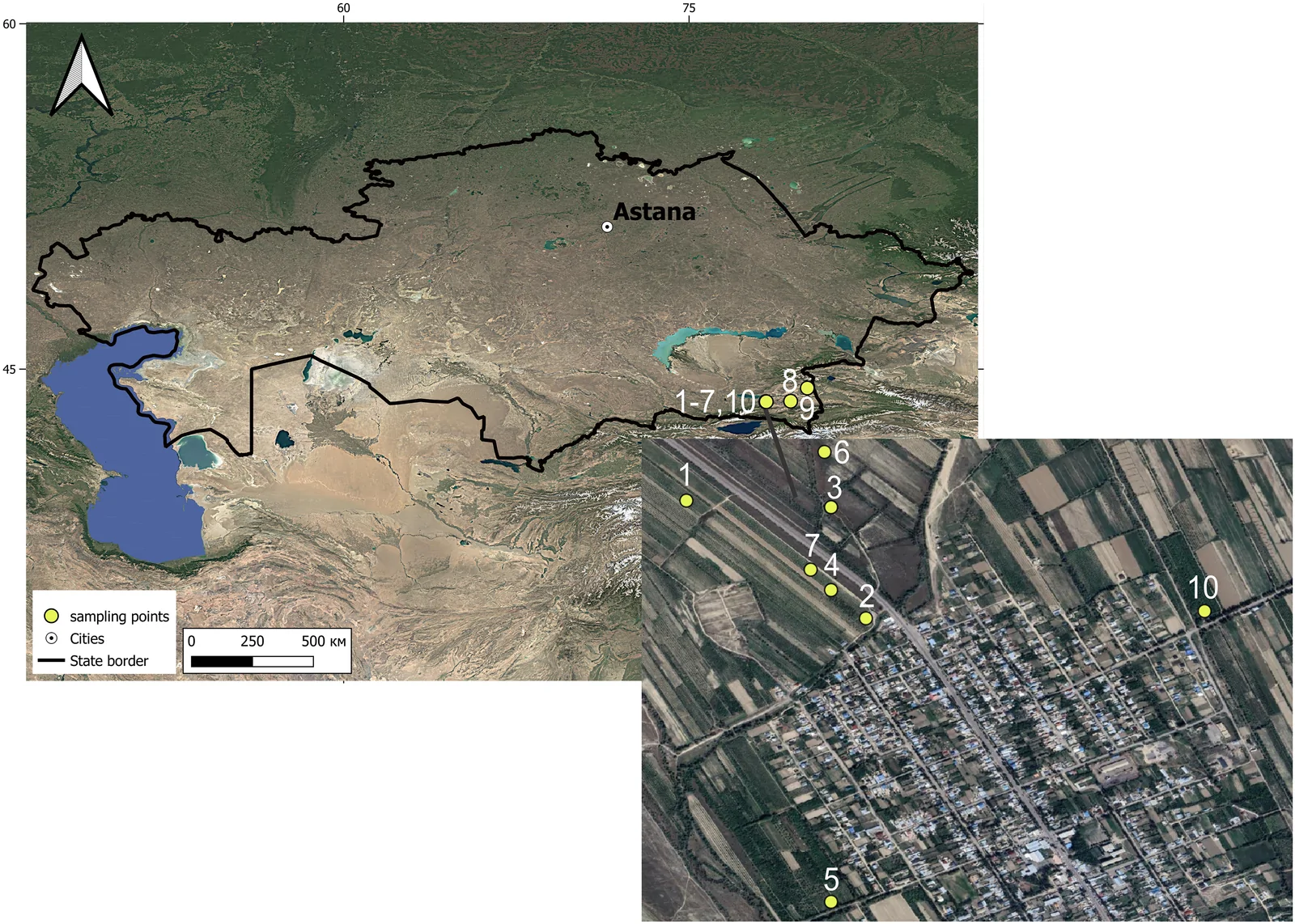
Map of the sampling sites. The enlarged inset shows a detailed view of sampling sites 1–7.

During the survey of apple orchards, *Comstockaspis perniciosa* was found on the bark of shoots and on the surface of apple fruits, where it formed colonies of varying densities. Under heavy infestations, the scale covers were closely aggregated, in some cases forming continuous colonies (**Figure 2A**). Infested fruits exhibited characteristic reddish-purple halos surrounding the feeding sites, a typical symptom of *C. perniciosa* damage (**Figures 2B, C**). The scale cover of the adult female was circular, slightly convex, greyish-brown in color, with well-defined centrally located yellow exuviae (**Figures 2E, F**). Removal of the scale cover revealed the soft body of the adult female, which was bright yellow and characteristically rounded in shape (**Figure 2H**). In addition to adult females, first-instar crawlers were observed. These exhibited an oblate body shape, bright yellow coloration, and lacked a protective scale cover (**Figure 2G**). The presence of the dispersal stage has indicated active population development during the survey period.

**Figure 2:**
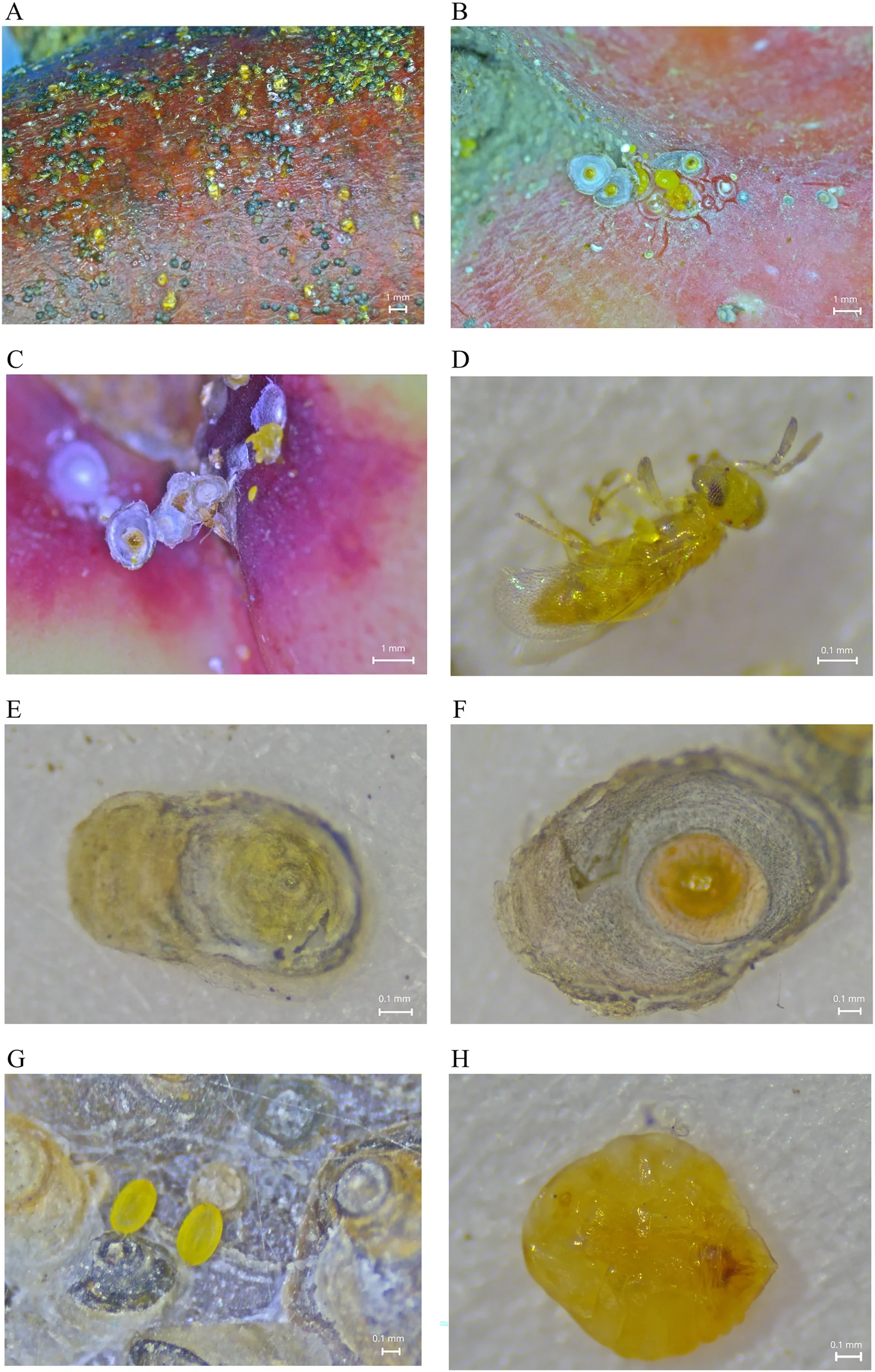
Morphological characteristics of *C. perniciosa*. **(A)** Heavy infestation of apple bark by *C. perniciosa*. **(B)** Colony of armored scales on the surface of an apple fruit. **(C)** Fruit damage showing characteristic reddish halos surrounding the feeding sites. **(D)** Adult male *C. perniciosa*. **(E, F)** Scale covers of adult *C. perniciosa*. **(G)** First-instar crawlers. **(H)** Adult female *C. perniciosa* after removal of the protective scale cover.

Taxonomic identification was subsequently confirmed by analysis of the nuclear 28S rRNA gene sequences. Analysis of the 28S rRNA gene sequences revealed that, in addition to consensus sequences of *Comstockaspis perniciosa*, a subset of the analyzed samples also contained sequences assigned to the family Aphelinidae. To clarify their taxonomic affiliation, reads were sequentially mapped against the reference databases of the families Diaspididae and Aphelinidae, after which the relative proportion of reads assigned to each family was determined.

Sequences belonging to the genus *Aphytis* were detected in ten *C. perniciosa* samples (**Figure 3C**) collected from the settlements of Bayseit and Bahar (sampling sites 1–8 in **Figure 1**). In all positive samples, the number of *Aphytis* reads was substantially lower than the number of *C. perniciosa* reads. This is most likely explained by the fact that DNA was extracted from *C. perniciosa* individuals, resulting in the majority of the recovered DNA originating from the host. In addition, parasitoids may have been present in the analyzed material only as eggs, larvae, pupae, or small tissue fragments beneath the scale cover, resulting in substantially lower biomass and, consequently, fewer sequencing reads than those derived from the scale insect.

**Figure 3:**
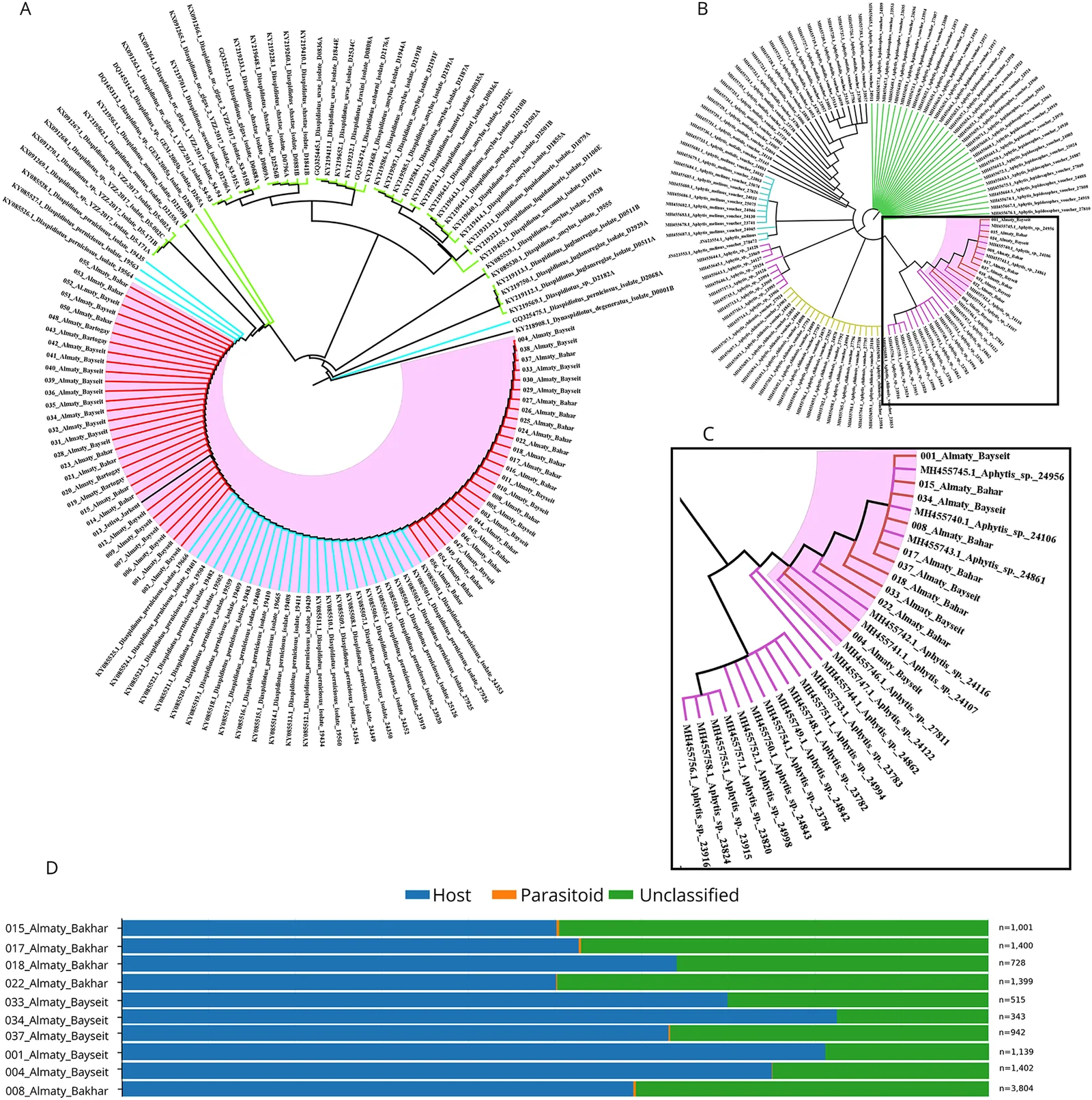
Phylogenetic analysis of 28S rRNA gene sequences. **(A)** Phylogenetic tree of 28S rRNA sequences of representatives of the family Diaspididae. The shaded sector indicates the *C. perniciosa* clade. **(B, C)** Phylogenetic tree of 28S rRNA sequences of representatives of the family Aphelinidae. The shaded sector indicates the *Aphytis* clade containing the sequences generated in the present study. **(D)** Relative proportions of sequencing reads assigned to Diaspididae (Host), Aphelinidae (Parasitoid), and the unclassified fraction (Unclassified). The total number of analysed reads (n) for each sample is shown on the right.

Phylogenetic analysis of the 28S rRNA gene sequences of *C. perniciosa* showed that all sequences generated in the present study clustered within a single well-supported clade together with reference *C. perniciosa* sequences retrieved from the NCBI database (**Figure 3A**). Sequences obtained from different localities in the Almaty Region exhibited a high degree of similarity and showed no evident geographic differentiation. These results confirm that all analyzed specimens belong to *C. perniciosa*. It should be noted that the analyzed region of the 28S rRNA gene is a highly conserved molecular marker widely used for taxonomic identification. Therefore, the absence of pronounced intraspecific variation is expected and indicates that this marker is not suitable for assessing population structure or inferring the origin of the studied populations.

A separate phylogenetic analysis was performed for the sequences identified as belonging to the genus *Aphytis* (**Figure 3B**). All sequences generated in the present study clustered within the *Aphytis* clade together with reference sequences, confirming their assignment to this genus. It should be noted that the reference sequences forming the same clade as those obtained in the present study are annotated in GenBank as *Aphytis* sp., without species-level identification. Consequently, more precise taxonomic identification of the detected parasitoids is not possible based on the currently available data. The 28S rRNA gene sequences obtained in this study were deposited in GenBank under accession numbers PZ785694–PZ785749 (*C. perniciosa*) and PZ780096–PZ780105 (*Aphytis* sp.).

## 4 Discussion

Over the past two decades, molecular methods have become one of the principal tools for the study of the family Diaspididae. Their widespread adoption has been driven by the limitations of traditional morphological diagnostics, which rely primarily on the characteristics of adult females, whereas males, immature developmental stages, and damaged specimens often lack sufficient diagnostic features for reliable identification (14, 16, 25). Additional challenges arise from the high degree of morphological conservatism and the presence of cryptic species (26). Consequently, molecular approaches have become an integral component of modern armored scale insect diagnostics.

The foundation of modern molecular systematics of Diaspididae was established by Andersen et al. (27), who reconstructed the phylogeny of the family using four molecular loci. Their study demonstrated the high utility of the 28S rRNA gene for resolving evolutionary relationships among armored scale insects. Subsequently, Schneider et al. (28) showed that many traditionally recognized genera, including *Diaspidiotus*, are not monophyletic, highlighting the limitations of classifications based solely on morphological characters. The results obtained in the present study are consistent with these findings: sequences from all *C. perniciosa* specimens formed a single clade together with reference sequences, confirming the suitability of the 28S rRNA marker for reliable taxonomic identification of armored scale insects. At the same time, the absence of intraspecific differentiation was expected, as the analyzed region is highly conserved and is not intended for population-level analyses.

Despite the worldwide distribution of *C. perniciosa*, molecular data for this species remain limited, and Central Asia is scarcely represented in international genetic databases. The sequences generated in the present study expand the known geographic representation of the species and may serve as a valuable resource for future studies of its distribution and origin.

The practical significance of this study is underscored by the fact that *C. perniciosa* is a quarantine pest included on the EPPO A2 List (29). The species was detected in commercial orchards located close to natural populations of *Malus sieversii*, the wild progenitor of the cultivated apple and an important source of genetic diversity for breeding programs (10, 30). The detection of active infestation foci and first-instar crawlers, which represent the only actively dispersing stage of the life cycle (3, 7, 25), indicates a potential risk of the pest spreading from cultivated orchards into natural ecosystems and emphasizes the need for phytosanitary monitoring of transition zones between agricultural and natural habitats.

Another important outcome of this study was the detection of sequences belonging to the genus *Aphytis* during the analysis of DNA extracted from scale insects. Members of this genus are specialized ectoparasitoids of Diaspididae and are widely used in biological control programs targeting *C. perniciosa* (31). Although the detection of parasitoids was not an objective of the study, the application of high-throughput sequencing enabled the simultaneous identification of both the phytophagous pest and its associated natural enemy. All detected sequences clustered confidently within the genus *Aphytis*; however, species-level identification was not possible because of the conserved nature of the 28S rRNA marker and the limited availability of reference sequences. These findings demonstrate the potential of NGS technologies for the simultaneous detection of quarantine pests and their natural enemies within phytosanitary monitoring programs.

## 5 Conclusions

As a result of the present study, populations of *Comstockaspis perniciosa* collected from commercial orchards in southeastern Kazakhstan were characterized based on external morphological observations and molecular data. Species-level identification was confirmed by 28S rRNA gene sequence analysis, and phylogenetic reconstruction demonstrated that all obtained sequences clustered with reference *C. perniciosa* sequences available in international databases. An important finding of this study was the detection of sequences belonging to the genus *Aphytis* in ten samples of *C. perniciosa*. Although species-level identification of the parasitoids was not possible due to the conserved nature of the analyzed 28S rRNA region and the limited availability of reference sequences, the results confirm the presence of representatives of this genus in the studied pest populations. The practical significance of these findings lies in the fact that the surveyed orchards were located in close proximity to natural populations of the wild apple species *Malus sieversii* and *M. niedzwetzkyana*, which are of exceptional conservation and genetic importance. The presence of established populations of this quarantine pest in such areas indicates a potential risk of its spread from cultivated orchards into natural ecosystems, highlighting the need for regular field monitoring of transition zones between agricultural and natural habitats and for the integration of molecular methods into early detection and quarantine pest surveillance programs.

## Data Availability

The datasets presented in this study can be found in online repositories. The names of the repository/repositories and accession number(s) can be found below: https://www.ncbi.nlm.nih.gov/genbank/, PZ785694–PZ785749 https://www.ncbi.nlm.nih.gov/genbank/, PZ780096–PZ780105.
